# Role of Vigorous Physical Activity in Mental Health: A Narrative Review

**DOI:** 10.7759/cureus.111007

**Published:** 2026-06-17

**Authors:** Konstantinos Stratakis, Jovana Todorovic, Zorica Terzić Šupić

**Affiliations:** 1 Institute of Medical Physiology, Faculty of Medicine, University of Belgrade, Belgrade, SRB; 2 Institute of Social Medicine, Faculty of Medicine, University of Belgrade, Belgrade, SRB

**Keywords:** anxiety, depressive symptoms, high-intensity interval training, mental health, mental well-being, perceived stress, physical activity, psychological distress, vigorous physical activity

## Abstract

Physical activity (PA) is widely associated with favorable mental health outcomes. However, much of the literature has examined PA as a broad exposure, usually in terms of total volume, participation status, or combined moderate-to-vigorous PA. This approach may neglect the potential importance of activity intensity. Vigorous physical activity (VPA) differs from lower-intensity activity in physiological demands, subjective effort, affective responses, and behavioral contexts and may, therefore, represent a distinct dimension of PA behavior. This narrative review aims to examine whether VPA should be considered a distinct dimension of PA behavior in mental health research by synthesizing mechanistic rationale, intervention evidence, and observational findings, while clarifying how differences in VPA definition, measurement approach, study design, population, and outcome selection contribute to inconsistent findings across the literature. The available evidence suggests that VPA is relevant to mental health, although the strength of evidence differs across outcomes. Findings are most developed for depressive symptoms, where both intervention and observational studies generally support favorable associations. Structured VPA may reduce depressive symptoms in some populations, while habitual VPA has been associated with lower depressive symptom burden in observational research. Evidence for other outcomes, including anxiety, perceived stress, psychological distress, sleep quality, and mental well-being, is more limited but suggests that the relevance of VPA may extend beyond depression alone. However, these findings remain less directly comparable because studies differ in outcome measures, populations, and study designs. Interpretation is complicated by the inconsistent definition of VPA across the literature. Prescribed VPA, habitual VPA, frequency of VPA, and the proportion of VPA within total PA are related but distinct constructs. High-intensity interval training is also a relevant, structured form of VPA, but it should not be treated as equivalent to all forms of VPA. These definitional differences, together with variation in measurement methods, populations, and mental health outcomes, help explain why findings remain inconsistent. Overall, VPA deserves consideration as a distinct feature of PA behavior in mental health research. Future studies should define VPA more precisely, distinguish absolute from proportion-based measures, account for total PA volume, and examine specific mental health outcomes separately.

## Introduction and background

In recent years, the relationship between physical activity (PA) and mental health has been the focus of extensive research. Across observational studies, prospective cohorts, and intervention research, higher levels of PA have generally been associated with more favorable mental health outcomes, including fewer depressive symptoms, lower anxiety, and reduced psychological distress [1-5]. This body of work has been important in establishing PA as a meaningful behavioral factor in mental health research. However, much of the literature treats PA as a broad construct, most commonly in terms of total activity volume, participation status, or combined moderate-to-vigorous PA (MVPA) [1-5]. Less attention has been paid to how activity is distributed across different intensity levels and to whether this distribution is relevant to mental health outcomes.

This distinction matters because the same total amount of PA can be achieved through varying intensity patterns. The World Health Organization recommends that adults engage in 150-300 minutes of moderate PA, 75-150 minutes of vigorous physical activity (VPA), or an equivalent combination of both [6]. Consequently, individuals may receive the same overall recommendation while engaging in substantially different activities. For example, one individual may accumulate activity primarily through moderate-intensity movement, whereas another may achieve a similar total volume predominantly through VPA. Therefore, similar total PA levels do not necessarily indicate equivalent PA patterns. This consideration is critical because the psychological and behavioral significance of PA may not be fully represented by total volume alone.

Interest in activity intensity has increased partly because evidence from studies examining physical health outcomes and PA intensity suggests that how activity is accumulated may matter, in addition to the total amount performed. Among middle-aged and older Australian adults, Gebel et al. found an inverse dose-response relationship between the proportion of VPA in total MVPA and all-cause mortality [7]. Similarly, Wang et al. reported in a large US cohort that, for the same total MVPA volume, a higher proportion of VPA was associated with lower all-cause mortality [8]. Although findings from studies on physical health cannot be directly transferred to mental health, they support the broader idea that PA should not always be treated as a single undifferentiated exposure. Intensity may reflect differences in physiological demand, perceived effort, behavioral preference, and the context in which activity occurs. A similar point has been raised in PA research across different contexts, where associations with mental health appear to vary according to whether activity occurs during leisure time, work, transport, or other behavioral contexts [5].

This issue is particularly relevant because the studies on mental health and PA have not addressed activity intensity with the same clarity as total PA volume. To address that question, it is necessary to critically assess the studies that have focused more directly on VPA. However, the literature on this topic is methodologically heterogeneous. Some intervention studies examine prescribed VPA as a structured exposure [9-12], whereas observational studies more often assess habitual VPA, VPA frequency, or the proportion of VPA within total PA [13-18]. Although these approaches are related, they are not equivalent.

This distinction is central to the present review. Absolute VPA refers to the total amount of VPA a person performs. In contrast, VPA proportion refers to how much of a person’s overall PA is vigorous rather than low or moderate. Therefore, someone may accumulate many minutes of VPA but still have a low VPA proportion if they also engage in large amounts of low or moderate PA. Conversely, someone with fewer total minutes of VPA may have a high VPA proportion if most of their activity is vigorous. Likewise, VPA prescribed in an intervention trial is not equivalent to habitual VPA measured in observational research. These distinctions are not solely technical; they may reflect different behavioral patterns, different measurement approaches, and potentially different relationships with mental health outcomes. This point is reinforced by the limited literature that has begun to examine the proportion of VPA in total PA more directly, with findings that are not fully consistent [15,16].

Therefore, this narrative review aims to examine VPA as a distinct dimension of PA behavior and its association with mental health. This article synthesizes selected evidence from broad PA and mental health reviews, VPA-focused intervention studies, and observational studies examining VPA as an absolute exposure, a frequency-based behavior, or a relative component of total PA. Relevant literature was identified through targeted searches of PubMed, Google Scholar, and the reference lists of key articles, with priority given to systematic reviews, meta-analyses, large observational studies, and studies directly examining VPA and its relation to mental health outcomes. The review first considers why VPA may be relevant to mental health, then summarizes the available evidence from intervention and observational studies, and finally discusses why findings in this field remain inconsistent. In doing so, it aims to clarify how VPA has been studied, how differences in definition and measurement affect interpretation, and what can currently be concluded from the available literature.

## Review

Mechanistic rationale for VPA and mental health

A central question in this review is whether VPA may have particular relevance to mental health beyond the broader effects already attributed to PA. At the conceptual level, this possibility is plausible because VPA differs from lower-intensity activity in physiological demand, subjective effort, and acute psychological experience [19]. These differences do not mean that VPA is uniformly superior to other forms of PA. However, they suggest that the mental health effects of PA may vary according to intensity and that VPA should not be treated only as part of a broader MVPA category.

One reason VPA may be relevant to mental health is its distinctive physiological effects. VPA typically involves greater metabolic demand than lower-intensity activity, meaning that a stronger physiological stimulus can be achieved within a shorter duration [20]. As intensity rises, this greater demand is accompanied by increased reliance on anaerobic metabolism and greater lactate accumulation [20].

Lactate has been proposed as a potential mediator of exercise-induced neuroplasticity, in part through its association with brain-derived neurotrophic factor (BDNF), a neurotrophin involved in synaptic plasticity and other processes relevant to brain function [21]. This does not establish lactate or BDNF as direct explanations for mental health benefits, but it provides a biologically plausible pathway through which PA intensity could influence brain-related outcomes. Evidence from studies examining high-intensity interval training (HIIT), one structured form of VPA, also suggests that VPA may influence reward-related brain systems. In healthy adults, Saanijoki et al. found that HIIT induced endogenous opioid release in several brain regions, a response that may be relevant to mood and reward processing [22]. Together, these findings provide a rationale for examining whether VPA has effects distinct from those of lower-intensity activity.

Beyond neuroplasticity and reward-related pathways, VPA may also be relevant because of its potential influence on stress-regulatory systems [23]. PA intensity appears to shape the body's response to subsequent psychological stress [23]. Caplin et al. found that PA intensity affected the cortisol response to a later psychosocial stressor, with higher-intensity PA associated with a more attenuated hypothalamic-pituitary-adrenal axis response [23]. This finding should not be interpreted as proof that VPA directly improves mental health outcomes, nor should cortisol responses be treated as direct proxies for subjective stress or anxiety. Rather, it suggests that VPA may interact with physiological systems involved in stress reactivity, while the psychological meaning of arousal may depend on appraisal, expectations, and context; similar physiological activation may be experienced as distressing, challenging, or energizing. This is relevant because stress regulation and stress appraisal are closely connected to several mental health outcomes, including depressive symptoms, anxiety, and psychological distress [24].

In addition to biological mechanisms, psychological processes may also help explain why VPA matters for mental health [25,26]. VPA often involves a stronger sense of effort, challenge, and exertion than lower-intensity movement. For some individuals, this may produce feelings of accomplishment, mastery, and confidence, which are relevant to self-efficacy and broader psychological functioning [25]. These processes may be especially important when VPA is performed voluntarily, progressively, and in a context that the individual experiences as meaningful or rewarding [26]. At the same time, VPA is unlikely to be experienced in the same way by all individuals. Affective responses to PA vary with intensity, with higher intensities more likely to elicit displeasure in some individuals, particularly when they exceed a personally tolerable threshold [26]. This variability is important because the effects of VPA on mental health may depend not only on its physiological intensity but also on whether the activity is experienced as rewarding, manageable, or aversive.

For these reasons, the mechanistic literature should be interpreted as supporting plausibility rather than providing direct evidence that VPA is superior to other forms of PA for mental health. Biological responses involving lactate, BDNF, endogenous opioids, and stress physiology may help explain why PA intensity may be important. In contrast, psychological responses involving effort, challenge, mastery, self-efficacy, and affective tolerance may influence how VPA is experienced.

Human evidence on VPA and mental health

The available literature includes both structured intervention studies, in which VPA is prescribed or delivered as part of an experimental protocol, and observational studies, in which VPA is assessed in habitual settings [9-18]. This distinction is important because prescribed VPA, HIIT, habitual VPA, VPA frequency, and the proportion of VPA within total PA are related but not interchangeable constructs.

Within the broader PA literature, review-level evidence has consistently shown that PA interventions are associated with improvements in depressive symptoms, anxiety, and psychological distress, with effects varying across PA modalities and participant characteristics [1,3]. However, these broader reviews examine PA interventions as a whole and do not, by themselves, determine whether VPA has a distinct role.

When attention is restricted to interventions specifically based on VPA, the evidence base becomes smaller and more concentrated on depressive symptoms than on other mental health outcomes [9-12]. The most direct synthesis currently available is a systematic review and meta-analysis of adolescents and young adults, which concluded that VPA interventions were associated with reductions in depressive symptoms [9]. This supports the idea that VPA may have mental health relevance in younger populations. At the same time, the available studies vary in intervention format, duration, supervision, comparator conditions, and participant characteristics, which limits firm conclusions about the optimal prescription of VPA for depressive symptoms [9].

Part of the intervention evidence also comes from the HIIT literature [10-12]. HIIT is relevant because it is one structured way of delivering VPA, but it should not be treated as equivalent to all forms of VPA. A systematic review and meta-analysis by Martland et al. reported that HIIT was associated with improvements in several mental health outcomes, including mental well-being, depression severity, psychological stress, and sleep disturbance, particularly when compared with non-active control conditions [10]. Evidence in the general population appears less consistent. Gaia et al. found no significant overall effect of HIIT on depressive or anxiety symptoms in healthy individuals and rated the certainty of evidence as very low [11]. By contrast, a recent meta-analysis focused on patients with depression reported modest improvements in depressive symptoms following high-intensity exercise, while also emphasizing small samples, heterogeneity across trials, and the need for larger randomized studies [12]. Taken together, these findings suggest that HIIT may be more likely to show measurable mental health effects in populations with more severe symptoms or diagnosed depression, but the evidence remains too heterogeneous to support broad conclusions about HIIT as a form of VPA or VPA in general as a uniform intervention [9-12].

Observational studies provide a more specific perspective on VPA as an everyday behavior, because they examine VPA as it is habitually accumulated rather than prescribed under experimental conditions. This evidence generally points toward favorable associations with mental health, particularly depressive symptoms, although the strength and interpretation of these associations vary according to how VPA is defined and measured [13,14]. In European adults aged over 65, Marques et al. reported that regular participation in MVPA was associated with lower depressive symptom severity in both cross-sectional and prospective analyses [13]. Using NHANES 2017-2020 data, Huang and Huang similarly found that greater VPA participation was associated with lower odds of depressive symptoms in US adults [14]. These findings suggest that habitual VPA may be associated with lower depressive symptom burden. However, they do not establish causality, as individuals who engage in VPA may differ from less active individuals in baseline health, fitness, socioeconomic position, and other health behaviors.

A more refined question is whether VPA matters because of the absolute amount performed or because it represents a larger share of the overall activity profile. Evidence on this point is less consistent [15,16]. Using NHANES 2007-2018 data, Yang et al. reported that a higher proportion of VPA within MVPA was associated with lower depression risk in some subgroups, particularly among men and middle-aged adults [15]. In contrast, an accelerometer-based analysis from the UK Biobank by Qiu and Xing found that a higher proportion of VPA within total PA was not associated with additional reductions in depressive symptoms once total PA volume was taken into account [16].

Evidence for mental health outcomes beyond depressive symptoms is more limited but suggests that VPA may also be relevant to broader psychological functioning [17]. Among college students, Van Kim and Nelson showed that more frequent VPA was associated with better mental health, lower perceived stress, and greater socializing [17]. In a multi-country study of university students, Pengpid and Peltzer reported that VPA was associated with lower depressive symptoms and perceived stress and better sleep quality [18]. These studies extend the observational evidence beyond depression alone, but they also reinforce the need for caution. Many studies use broad mental health indicators such as self-rated mental health, perceived stress, sleep quality, or social functioning rather than isolated symptom domains [27]. The studies on VPA and mental health are summarized in Table 1.

**Table 1:** Summary of human evidence on VPA and mental health HIIT: high-intensity interval training, MVPA: moderate-to-vigorous physical activity, PA: physical activity, VPA: vigorous physical activity

Evidence type	Main studies/reviews	How VPA was defined or studied	Main mental health outcomes	Main findings	Interpretation for this review
Broad PA/exercise intervention evidence	Singh et al., 2023 [1]; Noetel et al., 2024 [3]	Exercise or PA interventions broadly; not VPA-specific	Depression, anxiety, psychological distress	PA interventions generally improve mental health outcomes; depression effects vary by modality, population, and prescribed intensity.	Supports the general relevance of PA and exercise intensity, but does not isolate VPA as a distinct exposure.
VPA-specific intervention evidence	Yang et al., 2025 [9]	VPA interventions in adolescents and young adults	Depressive symptoms	VPA interventions were associated with reductions in depressive symptoms.	Provides the most direct intervention-level support for VPA, but evidence is concentrated on younger populations and depression.
HIIT/high-intensity intervention evidence	Martland et al., 2022 [10]; Gaia et al., 2024 [11]; Zeng and Wang, 2025 [12]	HIIT delivered as structured protocols	Depression, anxiety, stress, well-being, sleep	Findings are mixed: some reviews report improvements across mental health outcomes, evidence in healthy individuals is less consistent, and HIIT may show modest benefit in patients with depression.	HIIT is relevant as one structured form of VPA, but it should not be treated as equivalent to all VPA.
Observational evidence: habitual or absolute VPA	Marques et al., 2020 [13]; Huang and Huang, 2023 [14]	Regular participation in VPA or greater VPA participation	Depressive symptoms	Habitual VPA was generally associated with lower depressive symptoms.	Supports favorable associations between everyday VPA and depression-related outcomes, but causal inference remains limited.
Observational evidence: VPA frequency and broader mental health	VanKim and Nelson, 2013 [17]; Pengpid and Peltzer, 2018 [18]	Frequency of VPA or participation in VPA	Perceived stress, general mental health, sleep, socializing	More frequent VPA was associated with better general mental health indicators, lower stress, and better sleep quality and greater socializing.	Suggests VPA may be relevant beyond depression, but outcomes are broad and anxiety-specific evidence remains limited.
Observational evidence: proportion of VPA within total PA	Yang et al., 2022 [15]; Qiu and Xing, 2025 [16]	Relative contribution of VPA within MVPA or total PA	Depressive symptoms	Findings are inconsistent: proportion-based VPA was associated with lower depression risk in some NHANES subgroups, but not with additional benefit after total PA volume in UK Biobank accelerometer data.	Central to the review’s argument is the distinction between absolute and proportional VPA.

Sources of inconsistency in the VPA and mental health evidence

The evidence on VPA and mental health remains difficult to interpret as a single body. This inconsistency does not necessarily mean that the topic lacks relevance. Rather, it reflects the fact that studies often use similar terminology to examine different exposures, populations, outcomes, and research questions. In particular, findings vary depending on how VPA is defined, how activity intensity is measured, which mental health outcome is examined, and whether the study is designed to test a prescribed intervention or to describe habitual activity behavior.

A first source of inconsistency concerns the definition of the exposure itself. Studies that refer to VPA are not always studying the same construct. In intervention research, VPA may refer to prescribed VPA delivered under structured conditions [9], while HIIT represents one specific protocol-based form of VPA [10-12]. In observational research, VPA more often refers to habitual VPA, frequency of VPA, or the proportion of VPA within total PA or MVPA [13-18]. These constructs are related but not interchangeable. A study asking whether a person performs VPA several times per week is different from one asking whether VPA accounts for a larger share of that person’s total activity profile. Similarly, a supervised VPA intervention does not address the same question as a habitual observational study of habitual VPA. When these constructs are interpreted interchangeably, the literature can appear more contradictory than it actually is.

A second source of inconsistency concerns measurement. Distinguishing VPA from moderate or low activity requires either self-reported classification or device-based intensity estimation, and these approaches capture different aspects of behavior [28]. Self-report measures may reflect how individuals perceive and classify their own effort, but they are vulnerable to recall bias and differences in how participants interpret intensity categories [29]. Device-based measures reduce some limitations of self-report and can more consistently estimate movement intensity. However, they also depend on methodological decisions such as device placement, wear-time criteria, epoch length, and intensity cut-points [28]. They may also capture some forms of PA less accurately, including cycling, swimming, resistance exercise, and activities with limited wrist or hip movement [28]. This issue is especially important when the research question concerns the relative contribution of VPA rather than total PA volume alone. For example, Yang et al. used self-reported NHANES data. They found that a higher proportion of VPA within MVPA was associated with lower depression risk in some subgroups [15].

In contrast, Qiu and Xing used accelerometer-measured data from the UK Biobank. They found that a higher proportion of VPA within total PA was not associated with additional reductions in depressive symptoms after accounting for total PA volume [16]. These differences do not prove that one result is correct and the other is incorrect; rather, they show that measurement strategy and statistical modeling can shape conclusions about intensity-specific associations.

A third source of inconsistency concerns the mental health outcomes being examined. The clearest evidence for VPA appears to involve depressive symptoms, where both intervention and observational studies provide some support for favorable associations [9,12-16]. However, depression is only one mental health outcome, and mental health outcomes should not be treated as fully discrete or interchangeable categories. Other studies have examined anxiety, perceived stress, psychological distress, sleep quality, mental well-being, or broad mental health indicators [10,11,17,18]. These outcomes may overlap conceptually and symptomatically, but they are not equivalent. A finding for depressive symptoms should not be assumed to apply equally to anxiety, stress, sleep, or well-being, and a study using a broad mental health measure is not necessarily addressing the same question as a study focused on a specific symptom domain.

Population and context also matter. VPA may not have the same behavioral or psychological meaning across adolescents, university students, older adults, and general adult populations [9-18]. A VPA session may also be experienced differently depending on fitness, health status, prior activity experience, and motivation. When VPA is poorly matched to these individual factors, it may be experienced as excessive or aversive rather than beneficial. Injury, pain, excessive load, or negative prior experiences may reduce well-being and contribute to transient anxiety or depressive symptoms. Context is equally important, as VPA may occur through jogging, swimming, competitive sport, resistance training, HIIT, supervised intervention, leisure-time exercise, commuting, or occupational activity, each of which may differ in motivation, autonomy, social meaning, perceived reward, skill demands, injury risk, enjoyment, perceived control, and physiological load. This matters because research has shown that the relationship between PA and mental health varies according to the context in which activity occurs [5]. Therefore, inconsistent findings may partly reflect differences in who is performing VPA and under what circumstances.

Finally, observational studies can identify associations between habitual VPA and mental health outcomes, but they cannot establish causality or directionality [30]. Individuals who engage in different PA patterns differ in sex, age, field of study, and other contextual characteristics [31]. Better mental health may also make it easier for individuals to initiate and sustain VPA, rather than VPA being the sole cause of better mental health. Prospective designs and statistical adjustment can reduce some of these concerns, but they cannot remove them completely. This limitation is especially important when interpreting associations between habitual VPA and depressive symptoms or broader mental health indicators.

Taken together, inconsistencies in the VPA and mental health literature are best understood as problems of construct definition, measurement, outcome selection, population context, and causal interpretation. The evidence does not suggest that VPA should be dismissed as irrelevant. Instead, it shows that VPA should be studied with greater precision. Future research should specify whether it examines prescribed VPA, HIIT, habitual VPA, VPA frequency, or the proportion of VPA within total PA and align this definition with an appropriate measurement strategy and mental health outcome. Greater conceptual and methodological clarity is necessary before stronger conclusions can be drawn about the specific role of VPA in mental health.

## Conclusions

The available evidence suggests that VPA is relevant to mental health, especially depressive symptoms. However, it does not support a simple conclusion that vigorous intensity is uniformly superior to other forms of PA. Intervention studies suggest that structured VPA may reduce depressive symptoms in some groups, while observational studies generally support favorable associations between habitual VPA and mental health indicators. However, prescribed VPA, HIIT, habitual VPA, VPA frequency, and VPA proportion within total PA are distinct constructs and should not be interpreted interchangeably.

Future research should define VPA more precisely, distinguish structured from free-living VPA, separate absolute VPA from proportion-based measures, and account for total PA volume. Studies should also examine specific mental health outcomes separately and consider the context in which VPA occurs. Greater conceptual and methodological precision is needed to clarify whether VPA has independent relevance to mental health, for whom it may be most beneficial, and under which conditions it may support psychological well-being.
